# Left ventricular wall thickness in cats: agreement between echocardiographic views

**DOI:** 10.1093/jvimsj/aalaf084

**Published:** 2026-02-08

**Authors:** Jose Novo Matos, Virginia Luis Fuentes

**Affiliations:** Department of Veterinary Medicine, University of Cambridge, Cambridge CB3 0ES, United Kingdom; Clinical Science and Services, Royal Veterinary College, London AL9 7TA, United Kingdom

**Keywords:** feline, hypertrophic cardiomyopathy, cardiomyopathy

## Abstract

**Background:**

Various echocardiographic views are used to assess left ventricular wall thickness (LVWT), but whether these measurements are interchangeable remains unclear.

**Hypothesis/Objectives:**

To assess agreement of LVWT measurements between different echocardiographic views and techniques in cats.

**Animals:**

Four hundred eight cats: 292 with maximal LVWT (MaxLVWTd) < 6 mm and 116 with MaxLVWTd ≥6 mm.

**Methods:**

Cross-sectional study. Echocardiograms performed and measured by a single observer. Septal and free wall LVWT were measured using 2-dimensional (2D) right parasternal long-axis (RPLA) 4- and 5-chamber views, 2D short-axis (RPSA), and M-mode RPSA views. Bland–Altman analysis assessed agreement between views.

**Results:**

Septal thickness (4.6 mm [range, 2.9–9.5] vs 4.2 mm [range, 2.8–9.2], *P* < .0001) and free wall thickness (4.6 mm [range, 3.0–11.1] vs. 4.1 mm [range, 2.4–12.3]; *P* < .0001) were significantly greater in 2D RPLA than 2D RPSA. Agreement between 2D RPLA and 2D RPSA showed wide limits of agreement (LoA) and heteroscedasticity for septal and free wall measurements. Between 2D RPSA and M-mode RPSA, a small bias was noted for septal thickness (−0.04 mm, 95%, −0.12 to 0.04 mm), but LoA remained wide (−1.1 to 1.1 mm). Agreement between 4- and 5-chamber 2D RPLA showed small biases (septum: −0.09 mm, 95% CI −0.14 to −0.04 mm; free wall: −0.03 mm, 95% CI −0.09 to 0.02), with wide LoA (septum: −0.8 to 0.7 mm; free wall: −0.9 to 0.8 mm).

**Conclusions and clinical importance:**

LVWT measurements vary significantly across echocardiographic views and are not interchangeable. Standardized measurement protocols are needed to improve consistency in cardiac phenotyping.

## Introduction

Transthoracic echocardiography is the clinical gold standard for characterizing cardiac phenotype and diagnosing cardiomyopathies in cats.^1^ Hypertrophic cardiomyopathy (HCM) is the most common cardiac disease in cats. A clinical diagnosis of HCM is based on demonstration of increased left ventricular wall thickness (LVWT) using echocardiography.^1^^,^^2^ Reliable LVWT measurements are therefore essential for accurate phenotyping of cardiomyopathies.

In human medicine, the current echocardiographic guidelines for HCM recommend measuring LVWT using either a 2-dimensional (2D) parasternal long-axis^3^ or short-axis view.^4–6^ Assessment of all left ventricular (LV) segments from base to apex in a 2D short-axis view is preferable.^4^^,^^6^ When a long-axis view is used, cross-referencing with the short-axis view is recommended to help differentiate right ventricular (RV) structures from the compacted myocardium and to avoid tangential cuts through the septum.^3^ In contrast, no standardized veterinary echocardiographic guidelines currently exist for cats. Published studies of cardiomyopathy in cats indicate that clinicians use a variety of echocardiographic views and imaging techniques to assess LVWT, which can introduce variability in measurements. This variability can influence study results and limit the ability to compare findings with other studies. Limited data exist on whether LVWT measurements obtained from different echocardiographic views are interchangeable in cats.^7^

Measuring LVWT in cats presents specific challenges due to the small size of the cat heart and the presence of numerous intracavitary structures, such as trabeculations, LV bands (or false tendons), and papillary muscles, which should all be excluded from wall thickness assessments. Overestimation of LVWT occurs when imaging planes are off-axis or include these non-myocardial components. Some echocardiographic views and techniques might be more susceptible to off-axis image acquisition or inclusion of additional LV or RV structures, particularly when these structures are difficult to differentiate from the compacted myocardium. These and other technical factors could influence the agreement of LVWT measurements between different echocardiographic views.

The aim of this study was to assess the agreement of LV measurements obtained using different echocardiographic views and techniques in cats.

## Materials and methods

### Animals

Cross-sectional study. Echocardiograms performed and measured by a single operator (Jose Novo Matos) between May 2015 and March 2022 were included in the study. This was a convenience sample from 4 different studies.^8–11^ In accordance with our echocardiography laboratory protocol, cats included in this study had echocardiographic images recorded from all of the following views: a right-parasternal short-axis 2D view (2D RPSA) at the papillary muscle level, a right-parasternal long-axis 2D (2D RPLA) four-chamber (2D 4-Ch RPLA) and five-chamber (2D 5-Ch RPLA) views, and a 2D-guided M-mode of the left ventricle obtained from the RPSA view (M-mode RPSA). Cats with congenital heart disease, were excluded; there were no other exclusion criteria.

### Echocardiographic data

Echocardiographic examinations were performed using a Vivid E9 or Vivid I ultrasound machine (GE Systems, Hatfield, Hertfordshire, UK) with a 12 MHz phased-array transducer. A complete echocardiographic examination was performed in all cats including 2D, M-mode and Doppler studies from standard right- and left-sided views. Cats were gently restrained in lateral recumbency on a dedicated echocardiography table, and none were sedated for the examination. If a cat had > 1 echocardiogram stored over the study period, only the first echocardiogram was used. LVWT was measured at end-diastole at the thickest segment of the interventricular septum (IVSd) and of the free wall (LVFWd) using 3 different cardiac cycles (ie, 1 measurement of the thickest IVSd and LVFWd per cardiac cycle). Measurements were repeated in 3 2D image planes: 2D 4-Ch RPLA, 2D 5-Ch RPLA, and a 2D RPSA view. Measurements were also made in an M-mode RPSA view. The LVWT was measured with the aim of including only the compacted myocardium (excluding right ventricular structures, LV false tendons/bands, and papillary muscles),^3^^,^^12^ the observer used the ultrasound system’s cine function and carefully scrolled through the cardiac cycles to optimize visualization of the true compacted myocardium.^3^ The LVWT was measured using the leading-edge to leading-edge method over 3 cardiac cycles, and values were averaged. The LV internal dimension in end-diastole (LVIDd) was also measured in each of these views using the trailing edge to leading edge method (ie, at the blood-tissue interface) at the level of the chordae tendinea on 3 different cardiac cycles and averaged. End-diastole was defined as the echocardiographic frame showing the greatest LV internal diameter on 2D RPLA (after mitral valve closure) and RPSA views. The average IVSd, LVFWd, and LVIDd for each echocardiographic view was used for data analysis. The overall thickest LV segment (either IVSd or LVFWd) from each view was also used for data analysis and reported as maximal LVWT at end-diastole (MaxLVWTd). When comparing measurements from 2D RPLA with 2D RPSA views, the thickest segment of the IVSd and LVFWd (identified from either the 2D 4-Ch RPLA or 2D 5-Ch RPLA) was considered the 2D RPLA measurement.

All echocardiographic measurements were performed offline by a single board-certified veterinary cardiologist using a standard off-cart workstation (GE Echopac, GE Systems, Hatfield, Hertfordshire, UK). The measurements across the different echocardiographic views were acquired at the same time, without any specific order and the investigator was not blinded to the measurements obtained by each view/method. All measurements were performed at the time of the initial studies and were not repeated for the present study.

Echocardiographic images from 2D 4-Ch RPLA, 2D 5-Ch RPLA, 2D RPSA, and M-mode RPSA were obtained in all cats according to our laboratory protocol. However, not all measurements were available for every cat, as images considered oblique or containing fewer than 3 cardiac cycles at the time of acquisition were excluded and therefore not measured or analyzed. Consequently, data from every echocardiographic view were not available for each cat. The number of cats included in each analysis is reported in the Results section.

Data were analyzed separately in the whole cohort, in cats with LVWT < 6 mm, and in cats with LVWT ≥6 mm. The latter were defined as having a MaxLVWTd ≥6 mm in at least one echocardiographic view (ie, all views were considered when determining MaxLVWTd). Intra-observer repeatability and variability were determined by repeating LV measurements twice at least 4 weeks apart in a random sample of 10 studies (5 cats with MaxLVWT < 6.0 mm and 5 cats with MaxLVWT ≥6.0 mm).

### Clinical data

Clinical data collected included age, sex, body weight, body condition score (assessed on a scale of 1–9), systolic blood pressure and presence of clinical signs of cardiac disease. Systolic blood pressure was determined noninvasively using a Doppler device.^13^ In brief, measurements were obtained in a quiet environment using a cuff with a width of approximately 40% of the forelimb circumference. Five consecutive measurements were recorded and the mean value was used for analysis.

### Statistical analysis

Data were assessed for normality by both histogram analysis and a Shapiro–Wilk test. Parametric data are presented as mean (±SD) and non-parametric data presented as median (minimum–maximum). Categorical data is presented as frequency and percentage. A paired *t*-test or Wilcoxon signed-rank test was used (as appropriate) to compare LV measurements between echocardiographic views. Groups (ie, MaxLVWTd < 6 mm versus MaxLVWTd ≥6 mm) were compared using a Mann–Whitney U test for continuous non-parametric data and χ-square test for categorical data. The Holm-Sidak method was used to adjust the *P*-value for multiple comparisons. Bland–Altman analysis^14^ was used to assess agreement of LV measurements between echocardiographic views. Normal distribution of the differences between measurements was confirmed graphically and with a Shapiro–Wilk test. Linear regression was performed to determine if fixed or proportional bias existed. Agreement was assessed for the whole cohort, but also separately in cats with MaxLVWT < 6 mm and MaxLVWT ≥6 mm to determine the effect of LV hypertrophy on the level of agreement between methods. Bland–Altman analysis data is presented as average bias (95% CI) and 95% limits of agreement (LoA). We defined clinically relevant disagreement between echocardiographic views when the LoA between LVWT measurements were > 0.5 mm. Cohen’s κ was used to assess agreement of HCM diagnosis between different echocardiographic views. For this analysis, HCM phenotype was defined as MaxLVWTd ≥6 mm. Strength of agreement for HCM diagnosis (categorical variable) was defined in our study as follows: 0.41–0.60, moderate; 0.61–0.80 good; 0.81–1.0 excellent.^15^

Intra-observer repeatability and variability for LV measurements were quantified by intraclass correlation coefficient (ICC) and average percent coefficient of variation (CV), respectively. ICC estimates and their 95% CI were calculated based on a single-observer, absolute agreement, 2-way mixed-effects model. Percent CV was calculated as (SD of the measurements/average of the measurements) × 100. In our study, an ICC > 0.75 and CV < 10% were considered to indicate excellent measurement repeatability and low measurement variability, respectively.^16^

Statistical analysis was performed using commercially available software (GraphPad Prism version 10.4.1 for Mac OS X, GraphPad Software, San Diego, CA, USA; IBM SPSS Statistics for Mac, Version 29.0. Armonk, NY: IBM Corp). Bland–Altman analysis and plots were performed using an open-source web tool.^17^

## Results

We enrolled 408 cats, comprising 292 cats with MaxLVWTd < 6 mm and 116 cats with MaxLVWTd ≥6 mm. Study sample characteristics are shown in Table 1. The median age was 6.5 years (0.5–19 years). There were 204/408 (50%) male cats. The median body weight was 4.3 kg (2.1–8.4 kg) with a median body condition score of 5 (2–5). Non-pedigree cats were most common (178/408 cats, 43.6%), followed by Birman (152/408 cats, 37.3%) and Norwegian Forest cats (34/408 cats, 8.3%).

**Table 1:** Clinical characteristics of the study cohort.

Variable	All	MaxLVWTd < 6 mm	MaxLVWTd ≥6 mm	*P*-value
**Number of cats**	408	292	116	
**Age (years)**	6.5 (0.5–19)	5.9 (0.5–19)	**8.2** (0.8–15.6)	**.0005**
**Body weight (kg) (*n* = 352)**	4.3 (2.1–8.4)	3.9 (2.2–7.6)	**4.8** (2.1–8.4)	**<.0001**
**Sex: male *n*(%)**	204/408 (50)	119/292 (40.8)	**85/116 (73.3)**	**<.0001**
**SBP (mmHg) (*n* = 188)**	130 (60–260)	132 (90–260)	130 (60–190)	.6
**BCS (*n* = 342)**	5 (2–8)	5 (2–8)	5 (2–8)	.7
**Breed**				
** Nonpedigree *n*(%)**	178/408 (43.6)	95/292 (32.5)	**83/116 (71.6)**	**<.0001**
** Birman *n*(%)**	152/408 (37.3)	**145/292 (49.7)**	7/116 (6.0)	**<.0001**
** NFC *n*(%)**	34/408 (8.3)	**31/292 (10.6)**	3/116 (2.6)	**.008**
** Other *n*(%)**	45/408 (11.0)	21/292 (7.2)	**24/116 (20.7)**	**<.0001**
**Clinical signs *n*(%)**				
** Asymptomatic**		**288/292 (98.6)**	91/116 (78.4)	**<.0001**
** CHF**		1/292 (0.3)	**22/116 (19.0)**	**<.0001**
** ATE**		3/292 (1)	2/116 (1.7)	.6
** CHF + ATE**		–	1/116 (0.9)	
** Sudden death**		–	1/116 (0.9)	

Continuous variables are reported as median (range), and categorical variables as frequency (percentage). Mann–Whitney U test was used to compare continuous variables between cats with LVWT<6 mm and LVWT≥6 mm. χ-square was used for categorical variables. Statistically significant differences (*P* < .05) are highlighted in bold. Abbreviations: ATE = arterial thromboembolism; BCS = body condition score; CHF = congestive heart failure; LVH = left ventricular hypertrophy; NFC = Norwegian Forest cat; Other breeds = Bengal, British Shorthair, Burmese, Chinchilla, Maine Coon, Persian, Ragdoll, Russian White, Sphynx; SBP = systolic blood pressure.

### Echocardiographic data

Echocardiographic data are summarized in Tables 2–4.

**Table 2:** Left ventricular wall thickness and internal diameter at end-diastole measured using 2-dimensional long-axis and short-axis views in 368 cats.

Variable	2D RPLA	2D RPSA	*P*-value
**Whole cohort: 368 cats**			
** IVSd (mm)**	**4.6 (2.9–9.5)**	4.2 (2.8–9.2)	**<.0001**
** LVIDd (mm)**	15.1 (7.4–23.3)	15.3 (9.1–24.1)	.5
** LVFWd (mm)**	**4.6 (3.0–11.1)**	4.1 (2.4–12.3)	**<.0001**
** Max LVWTd (mm)**	**4.8 (3.3–11.1)**	4.4 (3.0–12.3)	**<.0001**
**MaxLVWTd ≥6 mm: 89 cats**			
** IVSd (mm)**	**6.4 (3.7–9.5)**	5.6 (3.4–9.2)	**<.0001**
** LVIDd (mm)**	15.0 ± 2.2	15.3 ± 2.1	.02
** LVFWd (mm)**	**6.0 (3.3–11.1**)	5.9 (3.4–12.3)	**.004**
** Max LVWTd (mm)**	**6.7 (4.7–11.1)**	6.1 (3.5–12.3)	**<.0001**
**MaxLVWTd < 6 mm: 279 cats**			
** IVSd (mm)**	**4.5 (2.9–5.9)**	3.9 (2.8–5.9)	**<.0001**
** LVIDd (mm)**	15.2 (10.7–23.3)	15.3 (10.9–24.1)	.4
** LVFWd (mm)**	**4.4 (3.0–5.9)**	3.9 (2.4–5.8)	**<.0001**
**Max LVWTd (mm)**	**4.6 (3.3–5.9)**	4.1 (3.0–5.9)	**<.0001**

Continuous variables are reported as median (range) or mean (±SD). Comparisons between long-axis and short-axis views were performed by Wilcoxon signed-rank test or paired *t*-test for nonnormally and normally distributed variables, respectively. Statistically significant differences are highlighted in bold, adjusted *P*-value (Holm-Sidak method): <.0127.

Abbreviations: 2D RPLA = 2-dimensional right parasternal long-axis view; 2D RPSA = 2-dimensional right parasternal short-axis view; IVS = interventricular septum at end-diastole; LVFWd = left ventricular free wall at end-diastole; LVIDd = left ventricular internal diameter at end-diastole; Max LVWTd = maximal end-diastolic left ventricular wall thickness.

**Table 3:** Left ventricular wall thickness and internal diameter at end-diastole measured using 2-dimensional 4-chamber and 5-chamber long-axis views in 242 cats.

Variable	2D 4-Ch RPLA	2D 5-Ch RPLA	*P*-value
**Whole cohort: 242 cats**			
** IVSd (mm)**	4.3 (2.6–7.6)	**4.5 (2.6–7.8)**	**<.0001**
** LVIDd (mm)**	14.7 (10.2–23.3)	15 (9.7–21.8)	.03
** LVFWd (mm)**	4.3 (2.6–8.0)	4.4 (3.0–7.9)	.3
** MaxLVWT (mm)**	4.5 (3.0–8.0)	**4.6 (3.0–7.9)**	**.003**
**MaxLVWT ≥6 mm: 34 cats**			
** IVSd (mm)**	6.0 (5.2–7.6)	6.4 (5.4–7.8)	.007
** LVIDd (mm)**	14.4 ± 2.2	14.9 ± 2.3	.1
** LVFWd (mm)**	5.6 (3.3–8.0)	5.5 (3.3–7.9)	.7
** Max LVWTd (mm)**	6.1 (5.2–8.0)	6.4 (5.4–7.9)	.1
**MaxLVWT < 6 mm: 208 cats**			
** IVSd (mm)**	4.2 (2.6–5.9)	**4.3 (2.6–5.9)**	**.0007**
** LVIDd (mm)**	14.8 (10.7–23.3)	15 (10.6–21.8)	.09
** LVFWd (mm)**	4.2 ± 0.5	4.2 ± 0.5	.3
** Max LVWTd (mm)**	4.4 (3.0–5.9)	4.5 (3.0–5.9)	.009

Continuous variables are reported as median (range) or mean (±SD). Comparisons between long-axis 4 chamber and long-axis 5 chamber views were performed by Wilcoxon signed-rank test or paired *t*-test for nonnormally and normally distributed variables, respectively. Statistically significant differences are highlighted in bold, adjusted *P*-value (Holm-Sidak method): <.005. Abbreviations: 4-CH RPLA = four chamber right parasternal long-axis view; 5-CH RPLA = five chamber right parasternal long-axis view; IVS = interventricular septum at end-diastole; LVFWd = left ventricular free wall at end-diastole; LVIDd = left ventricular internal diameter at end-diastole; Max LVWTd = maximal end-diastolic left ventricular wall thickness.

**Table 4:** Left ventricular wall thickness and internal diameter at end-diastole measured using 2-dimensional and M-mode images acquired from the right parasternal short-axis view in 200 cats.

Variable	2D RPSA	M-Mode RPSA	*P*-value
**Whole cohort: 200 cats**			
** IVSd (mm)**	4.1 (2.8–9.2)	4.2 (2.6–8.1)	.2
** LVIDd (mm)**	15.4 ± 1.8	15.5 ± 1.8	.8
** LVFWd (mm)**	4.1 (2.4–9.3)	**4.2 (2.5–9.2)**	**.0001**
** Max LVWTd (mm)**	4.2 (3.0–9.3)	**4.4 (2.7–9.2)**	**.0003**
**MaxLVWT ≥6 mm: 38 cats**			
** IVSd (mm)**	5.7 ± 1.1	5.7 ± 1.1	.8
** LVIDd (mm)**	15.7 (9.1–19.9)	15.7 (9.9–19.6)	.9
** LVFWd (mm)**	5.8 (4.0–9.3)	6.2 (3.9–9.2)	.5
** Max LVWTd (mm)**	6.3 ± 1.3	6.5 ± 1.2	.2
**MaxLVWT < 6 mm: 162 cats**			
** IVSd (mm)**	4.0 ± 0.5	4.0 ± 0.6	.2
** LVIDd (mm)**	15.4 ± 1.7	15.4 ± 1.7	.9
** LVFWd (mm)**	3.9 ± 0.5	**4.1 ± 0.6**	**<.0001**
** Max LVWTd (mm)**	4.1 (3.0–5.9)	**4.3 (2.7–5.7)**	**.0003**

Continuous variables are reported as median (range) or mean (±SD). Comparisons between 2 dimensional and M-mode short-axis views were performed by Wilcoxon signed-rank test or paired *t*-test for nonnormally and normally distributed variables, respectively. Statistically significant differences are highlighted in bold, adjusted *P*-value (Holm-Sidak method): <.0057. Abbreviations: 2D RPSA = 2-dimensional right parasternal short-axis view: M-mode of the left ventricle acquired in RPSA view; IVS = interventricular septum at end-diastole; LVFWd = left ventricular free wall at end-diastole; LVIDd = left ventricular internal diameter at end-diastole; Max LVWTd = maximal end-diastolic left ventricular wall thickness.

### LV measurements in 2D RPLA versus 2D RPSA

Left ventricular measurements in both 2D RPLA (either 2D 4Ch- RPLA or 2D 5Ch-RPLA) and 2D RPSA were available in 368 cats (Table 2). The LVWT measurements for both IVSd and LVFWd were significantly greater in 2D RPLA than 2D RPSA across the whole study sample, as well as in cats with MaxLVWTd < 6 and in cats with MaxLVWTd ≥6 mm. The absolute mean of the difference between 2D RPLA and 2D RPSA for IVSd, LVFWd, and MaxLVWTd measurements was 0.66 mm (95%CI, 0.59–0.72 mm), 0.55 mm (95% CI, 0.50–0.59 mm), and 0.59 mm (95% CI, 0.54–0.65 mm), respectively. The LVIDd measurements were similar between these echocardiographic views.

**Figure 1:**
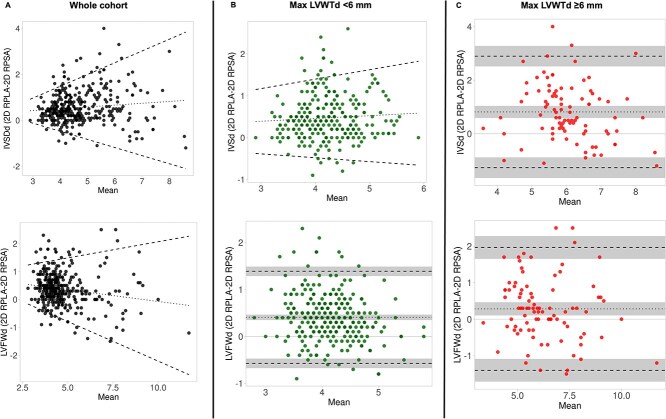
Bland–Altman plots assessing agreement between left ventricular wall thickness (LVWT) measurements obtained from 2-dimensional right parasternal long-axis (2D RPLA) and short-axis (2D RPSA) views in (A) the whole cohort, (B) cats with maximal LVWT <6 mm, and (C) cats with maximal LVWT ≥6 mm.

Evaluation of agreement between 2D RPLA and 2D RPSA in the whole cohort showed heteroscedasticity for both the IVSd and LVFWd measurements (Figure 1A), where the degree of disagreement increased (wider LoA) with increasing LVWT. There was a slight proportional bias for the IVSd, where measurements acquired in 2D RPLA were progressively larger than measurements in 2D RPSA at thicker LV walls. There was also a slight proportional bias for the LVFWd, but in this case the measurements in 2D RPLA were smaller than in 2D RPSA at thicker LV walls. When we assessed only the cats with MaxLVWTd < 6 mm, there was also greater disagreement (wider LoA) for the IVSd measurements between 2D RPLA and 2D RPSA at increasing LVWT (heteroscedasticity; Figure 1B). The LVFWd measurements were greater in 2D RPLA than 2D RPSA with a fixed bias of 0.41 mm (95% CI 0.35–0.47 mm) and wide limits of agreement (95% LoA −0.57 to 1.39 mm) (Figure 1B). In cats with thicker LV walls (MaxLVWTd ≥6 mm), both the IVSd and LVFWd measurements were greater in 2D RPLA than 2D RPSA with fixed biases and wide limits of agreement (IVSd bias 0.81 mm (95% CI 0.59–1.0 mm), 95% LoA −1.3 to 2.9 mm; LVFWd bias 0.3 mm (95% CI 0.1–0.5 mm), 95% LoA –1.4 to 2.0 mm) (Figure 1C). There was a clinically relevant disagreement on LVWT measurements between 2D RPLA and 2D RPSA, indicating that the 2 views are not interchangeable for measuring LVWT. Conversely, LVIDd showed small, fixed biases, and narrow LoA between 2D RPLA and 2D RPSA across the study groups (whole cohort, bias −0.09 mm (95%CI −0.23 to 0.05 mm), 95% LoA −2.8 to 2.6 mm; cats with LVWT≥6 mm, bias −0.46 mm (95% CI −0.82 to −0.09 mm), 95% LoA −3.86 to 2.9 mm) suggesting that LVIDd can be measured interchangeably between these 2 echocardiographic views.

### LV measurements in 2D 4-Ch RPLA versus 2D 5-Ch RPLA

We had 242 cats with LV measurements acquired in both 2D 4Ch- RPLA and 2D 5Ch-RPLA (Table 3). The IVSd measurements were significantly greater in 2D 5Ch-RPLA in the whole cohort and in cats with MaxLVWTd < 6 mm. The LVFWd and LVIDd were similar between echocardiographic views across the study groups. The absolute mean of the difference between 2D 4-Ch RPLA and 2D 5-Ch RPLA for IVSd, LVFWd, and MaxLVWTd measurements was 0.31 mm (95% CI, 0.27–0.35 mm), 0.31 mm (95% CI, 0.28–0.35 mm), and 0.29 mm (95% CI, 0.25–0.33 mm), respectively.

**Figure 2:**
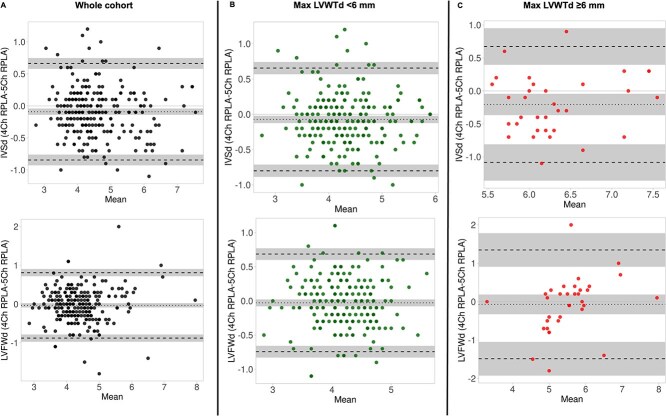
Bland–Altman plots assessing agreement between left ventricular wall thickness (LVWT) measurements obtained from 2-dimensional right parasternal 4-chamber (2D 4-Ch RPLA) and 5-chamber (2D 5-Ch RPLA) long-axis views in (A) the whole cohort, (B) cats with maximal LVWT <6 mm, and (C) cats with maximal LVWT ≥6 mm.

Assessment of agreement between 2D 4Ch-RPLA and 2D 5Ch-RPLA views in the whole cohort showed a small, fixed bias for both the IVSd (−0.09 mm, 95% CI, −0.14 to −0.04 mm) and LVFWd (−0.03 mm, 95% CI, −0.09 to 0.02) with wide LoA (IVSd 95% LoA −0.8 to 0.7 mm, LVFWd 95% LoA −0.9 to 0.8 mm). Similarly, there were small, fixed biases with wide LoA for both the IVSd and LVFWd in the study groups (MaxLVWTd < 6 mm and MaxLVWTd ≥6 mm) (Figure 2B and C). There was a clinically relevant disagreement on LVWT measurements between 2D 4Ch-RPLA and 2D 5Ch-RPLA, indicating that the 2 views are not interchangeable for measuring LVWT. Conversely, LVIDd showed small, fixed biases, and narrow LoA across the study groups suggesting that LVIDd can be measured interchangeably between these 2 echocardiographic views (whole cohort, bias −0.15 mm (95%CI, −0.3 to 0.01 mm), 95% LoA −2.4 to 2.1 mm; cats with MaxLVWT<6 mm, bias −0.1 mm (95% CI, −0.23 to 0.04 mm), 95% LoA −2.0 to 1.8 mm); cats with LVWT≥6 mm, bias −0.5 mm (95% CI, −1.2 to −0.15 mm), 95% LoA −4.13 to 3.1 mm.

### LV measurements in 2D RPSA versus M-mode RPSA

We had 200 cats with LV measurements acquired in both 2D RPSA and M-mode RPSA (Table 4). The LVFWd measurements were significantly greater in M-mode RPSA in the whole cohort and in cats with MaxLVWTd < 6 mm. The IVSd and LVIDd were similar between echocardiographic views across the study groups. The absolute mean of the difference between 2D RPSA and M-mode RPSA for IVSd, LVFWd, and MaxLVWTd measurements was 0.42 mm (95% CI, 0.36−0.47 mm), 0.47 mm (95% CI, 0.41 to 0.54 mm), and 0.46 mm (95% CI, 0.40–0.52 mm), respectively.

**Figure 3:**
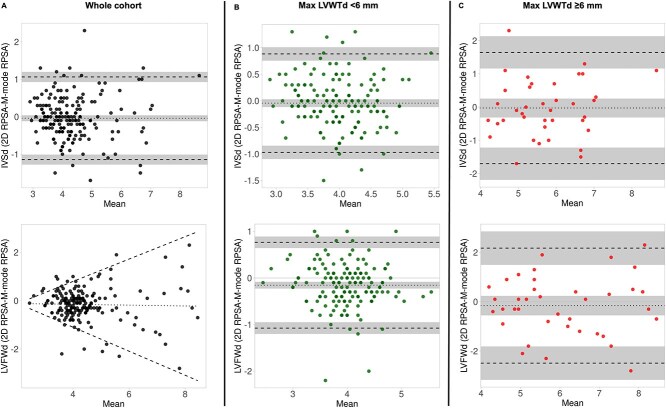
Bland–Altman plots evaluating agreement between left ventricular wall thickness (LVWT) measurements obtained from 2-dimensional (2D) and M-mode images acquired in the right parasternal short-axis (RPSA) view in (A) the whole cohort, (B) cats with maximal LVWT <6 mm, and (C) cats with maximal LVWT ≥6 mm.

In the whole cohort, there was a small, fixed bias (−0.04 mm, 95% CI, −0.12 to 0.04 mm) between 2D-RPSA and MMode-RPSA with wide 95% LoA for the IVSd (95% LoA −1.1 to 1.1 mm), and with heteroscedasticity (increased disagreement with increasing LVWT) for the LVFWd (Figure 3A). When we looked separately at cats with thinner (MaxLVWTd < 6 mm) and thicker (MaxLVWTd ≥6 mm) LV walls there were small, fixed biases for both the IVSd and LVFWd with wide LoA (cats with MaxLVWT<6 mm, IVSd bias −0.04 mm (95% CI, −0.12 to 0.03), 95% LoA −0.97 to 0.88 mm; LVFWd −0.15 mm (95%CI, −0.23 to −0.08 mm), 95% LoA −1.1 to .77 mm; cats with MaxLVWT≥6 mm, bias −0.03 (95% CI, −0.31 to 0.26 mm), 95% LoA −1.7 to 1.6 mm; LVFWd bias −16 mm (95% CI, −0.55 to 0.24 mm), 95% LoA −2.5 to 2.2 mm) (Figure 3B and C). These reflect a clinically relevant disagreement on LVWT measurements between 2D and M-mode measurements obtained from a short-axis view. Similarly to the other echocardiographic views, LVIDd measured in 2D RPSA and MMode RPSA showed small, fixed biases, and narrow LoA suggesting a good agreement between these 2 techniques (whole cohort, bias −0.02 mm (95%CI, −0.17 to 0.13 mm), 95% LoA −2.2 to 2.1 mm; cats with MaxLVWT<6 mm, bias −0.01 mm (95% CI, −0.17 to 0.14 mm), 95% LoA −1.98 to 1.96 mm); cats with LVWT≥6 mm, bias −0.04 mm (95% CI, −0.51 to 0.42 mm), 95% LoA −2.8 to 2.7 mm.

There was only a moderate to good agreement in HCM diagnosis between echocardiographic views (2D RPLA versus 2D RPSA κ 0.66 (95%CI, 0.56−0.76); 2D 4-Ch RPLA versus 5-Ch RPLA κ 0.74 (0.60–0.88); 2D RPSA versus M-mode RPSA κ 0.71 (95% CI, 0.55–0.87)).

Intra-observer repeatability was excellent with low measurement variability for MaxLVWTd (ICC 0.96 (95%CI, 0.64–0.99), %CV 5% (95%CI, 2–8)) and LVIDd (ICC 0.99 (95%CI, 0.97–0.99), %CV 1.3% (95%CI, 0.8–1.7)).

## Discussion

In this study, we investigated the agreement of LVWT measurements obtained from different echocardiographic views and techniques in cats. The results indicate that LVWT measurements are not interchangeable between views, as evidenced by wide and clinically relevant LoA. Such variability in LVWT measurements might influence cardiac phenotyping and subsequent clinical decision-making. In contrast, measurements of LVIDd demonstrated good agreement across views, suggesting that different echocardiographic views can be used interchangeably for the assessment of this variable.

Our findings align with those of a previous, smaller study, demonstrating that LVWT measurements obtained using 2D and M-mode echocardiography in cats are not interchangeable, as these modalities yielded significantly different values that affected the classification of LVH.^7^ Our study corroborates these earlier results and further extends them by demonstrating that different 2D right parasternal echocardiographic views are also not interchangeable and could produce clinically relevant differences in LVWT measurements, potentially influencing the diagnosis of HCM.

Current human echocardiographic guidelines for the assessment of HCM recommend measuring LVWT using either the 2D RPLA^3^ or the 2D RPSA view.^4^^,^^5^ It is essential that LVWT measurements exclude papillary muscles and right ventricular structures, including trabeculations, the moderator band, crista supraventricularis, and components of the tricuspid valve apparatus, as inclusion of these structures might result in overestimation of wall thickness and subsequent misdiagnosis of HCM.^3^^,^^5^^,^^18^ Accordingly, only the compact myocardium should be included in LVWT measurements.^3^ In our study, we aimed to measure only the compact myocardium; however, the inadvertent inclusion of LV or RV structures can vary depending on the imaging view and might account for discrepancies in LV wall thickness measurements across views. Even in humans, with larger hearts, differentiating the true compact myocardium from adjacent LV and RV structures using standard echocardiography can be challenging.^3^

In people, measurements obtained from long-axis views can overestimate LVWT due to the potential for tangential sectioning of the LV wall and the inclusion of those structures adjacent to, but not part of, the compact myocardium.^18^ Consequently, 2D short-axis views are suggested by some as more accurate for the quantification of LVWT.^4^^,^^6^^,^^18^ Nevertheless, comprehensive evaluation across all available imaging planes is advised to confirm LVWT, and measurements obtained in long- and short-axis views at corresponding anatomical levels should demonstrate concordance.^3^^,^^4^

In our study, the marked differences in LVWT measurements between echocardiographic views and techniques might be explained, as described in human studies, by slight tangential cuts through the LV wall (which are difficult to detect without biplane imaging) or by failure to correctly identify the true compacted myocardium in certain LV segments.^3^ The LVWT measurements obtained from the 2D RPLA view were consistently greater than those from the 2D RPSA view. This discrepancy might result from subtle tangential imaging of the LV wall or inadvertent inclusion of right ventricular trabeculations and papillary muscles, as reported in human echocardiographic studies. Anecdotally, delineation of the compact myocardium and identification of adjacent right ventricular structures are more readily achieved using the 2D RPSA view, which are described has an excellent view to differentiate right ventricular structures from compacted myocardium.^3^ Furthermore, RPSA views might make it easier to recognize off-axis imaging planes, thereby reducing the risk of tangential LVWT measurements.^19^ Finally, the differences between views might also reflect measurement of different LV segments.

Left ventricular bands (also known as false tendons) are common in cats, and some can run parallel to the LV wall, making them difficult to detect on echocardiography.^20^ These structures can also potentially contribute to overestimation of LVWT in certain views where their identification is more challenging.

The LoA for LVWT measurements between different echocardiographic views were wide and exceeded the 5–6 mm range commonly used by various authors to define normal LVWT. Consequently, the choice of echocardiographic view can influence the echocardiographic diagnosis (ie, cardiac phenotyping). This is reflected by only moderate to good agreement between views in diagnosing HCM in our study. For different echocardiographic views to be used interchangeably, the agreement between views would need to be excellent, with narrow LoA. Currently, there are no standardized veterinary echocardiographic guidelines for cats, and studies on cardiomyopathies in cats often use different views to measure LVWT. This variability can affect study results, limiting the comparability of findings across studies, and potentially affecting clinical trial results.

In contrast to LVWT measurements, LVIDd measurements were consistent across views and demonstrated good agreement. This supports the expectation that LVIDd can be reliably obtained using different echocardiographic views and techniques. Similar findings are reported in dogs.^21^ The consistency of LVIDd across views in our study also reinforces the hypothesis that discrepancies in LVWT might result from the variable and erroneous inclusion of extra-mural structures attached to the LV walls in the thickness measurements or from measurements obtained from different wall segments.

Our study has several limitations. Left ventricular measurements were not available in every cat for all echocardiographic views, as images deemed oblique or with inadequately recorded video loops were excluded. Additionally, the investigator performing the measurements was not blinded to the values obtained from each view, which could have introduced measurement bias. However, any potential bias would likely have reduced variability and falsely improved agreement between views. Since poor agreement was observed, this bias is unlikely to have influenced the main findings. The study was conducted at a single center, with all echocardiograms and measurements performed by a single investigator. While this reduces operator variability, it may introduce bias related to the operator’s technique and experience. Future studies using multiple blinded echocardiographers would provide important additional insights into the level of agreement between views. Furthermore, LV measurements were not timed using an ECG, which could have led to inconsistencies in identifying end-diastole across views. Therefore, we cannot determine whether ECG-timed measurements would have resulted in better agreement.

In conclusion, our study suggests that LVWT measurements obtained from different echocardiographic views are not interchangeable due to the wide LoA observed. This variability can influence cardiac phenotyping in cats. These findings underscore the need for standardized echocardiographic guidelines in cats.

## Off-label antimicrobial declaration

Authors declare no off-label use of antimicrobials.

## Institutional animal care and use committee or other approval declaration

This study was approved by the Animal Ethics and Welfare Committee of the Department of Veterinary Medicine, University of Cambridge (CR704).

## Abbreviations

2D: 2 dimensional
4-Ch RPLA: four chamber right parasternal long-axis view
5-Ch RPLA: five chamber right parasternal long-axis view
CI: confidence interval
CV: coefficient of variation
HCM: hypertrophic cardiomyopathy
ICC: intraclass correlation coefficient
IVSd: interventricular septum in end-diastole
LoA: limits of agreement
LV: left ventricle
LVFWd: left ventricular free wall in end-diastole
LVIDd: left ventricular internal dimension in end-diastole
LVWT: left ventricular wall thickness
MaxLVWT: maximal left ventricular wall thickness
RPLA: right parasternal long-axis view
RPSA: right parasternal short-axis view
